# Contribution of magnetic resonance imaging to the prenatal diagnosis of common congenital vascular anomalies

**DOI:** 10.1007/s00247-021-05031-w

**Published:** 2021-04-23

**Authors:** Laurence Crivelli, Anne-Elodie Millischer, Pascale Sonigo, David Grévent, Sylviane Hanquinet, Yvan Vial, Leonor Alamo

**Affiliations:** 1 Department of Diagnostic and Interventional Radiology, CHUV – Centre Hospitalier Universitaire Vaudois, Rue du Bugnon 21, 1011 Lausanne, Switzerland; 2 Department of Pediatric Radiology, Hôpital Universitaire Necker - Enfants Malades, Paris, France; 3 Department of Radiology, HUG – Hôpitaux Universitaires de Genève, Genève, Switzerland; 4 Department of Gynecology and Obstetrics, CHUV – Centre Hospitalier Universitaire Vaudois, Lausanne, Switzerland

**Keywords:** Congenital hemangiomas, Fetus, Lymphatic malformations, Magnetic resonance imaging, Prenatal diagnosis, Ultrasound, Vascular anomalies

## Abstract

**Background:**

Screening ultrasound (US) has increased the detection of congenital vascular anomalies in utero. Complementary magnetic resonance imaging (MRI) may improve the diagnosis, but its real utility is still not well established.

**Objectives:**

We aimed to describe the imaging findings on prenatal US and MRI of the most frequent congenital vascular anomalies (lymphatic malformations and congenital hemangiomas) to assess the accuracy of prenatal US and MRI exams for diagnosis and to evaluate the relevance of the additional information obtained by complementary fetal MRI.

**Materials and methods:**

All confirmed postnatal congenital vascular anomalies detected in the last 10 years at 3 university hospitals were retrospectively identified. The prenatal diagnosis was compared with the final diagnosis for both methods and the clinical relevance of additional MRI information was evaluated. A second MRI in advanced pregnancy was performed in fetuses with lesions in a sensitive anatomical location and the clinical relevance of the additional information was evaluated.

**Results:**

Twenty-four cases were included in the study, 20 lymphatic malformations and 4 hemangiomas. MRI slightly improved the diagnosis of lymphatic malformation, 85% vs. 80% at US, especially for abdominal lesions. Both methods had a low identification rate (25%) for tumors. MRI performed late in five fetuses with lymphatic malformation allowed optimized management at birth.

**Conclusion:**

MRI improves the diagnosis of congenital lymphatic malformations whereas hemangiomas remain difficult to identify in utero. The main role of MRI is to provide high-defined anatomical data to guide management at birth.

## Introduction

After decades of confusing nomenclature, the International Society for the Study of Vascular Anomalies (ISSVA) adopted in 1996 the classification suggested by Mullicken and Glowacki in 1982 [1], which distinguishes between:

1. Vascular malformations, which are developmental alterations of vascular channels. They are classified according to the predominant type of vessel in the malformation into arterial, venous, capillary, lymphatic or mixed [2, 3], and have a characteristic high- or low-flow pattern in the presence or absence of an arterial component [1, 2]. They may be part of an “overgrowth disorder” in a wide variety of syndromes that combine a vascular malformations with a dysplastic, enlarged bone, limb or even a body-half [3, 4].
2. Vascular tumors, which are formed by proliferative and hyperplastic endothelial lesions and typically have a high-flow arterial pattern. In contrast to the most common vascular tumor, benign infantile hemangioma, congenital hemangiomas are fully developed at birth. For a long time, vascular tumors were classified according to their postnatal clinical evolution into rapidly involuting congenital hemangiomas (RICH), which have a spontaneous and complete resolution without therapy in <14 months, and non-involuting congenital hemangiomas (NICH), which grow with the child, show no regression and may require therapy [2, 4, 5]. More recently, partially involuting hemangiomas (PICH) have been described as an intermediate type that initially behaves as a RICH lasting as a NICH [6].

The generalization of screening ultrasound (US) during pregnancy has increased the rate of detection of vascular anomalies in utero [7, 8]. An accurate prenatal diagnosis may help the medical team evaluate the extension and precise anatomical location of these lesions and improve the information provided to the parents, including predictions regarding prognosis during pregnancy, at birth and in the neonatal period [2, 9].

Prenatal magnetic resonance imaging (MRI) can be a complementary imaging method for fetal pathology, but its usefulness in patients with congenital vascular anomalies is not well known. The aims of this study are to describe the prenatal imaging findings on US and MRI of the two most frequently detected congenital vascular anomalies: lymphatic malformations and congenital hemangiomas. We compare the accuracy of both methods for diagnosis and evaluate the relevance of the additional information provided by complementary prenatal MRI for management.

## Materials and methods

### Inclusion criteria

A retrospective chart review of a 10-year period (January 2010 to December 2019) was conducted for all neonates with a confirmed congenital lymphatic malformation or hemangioma with available prenatal US and MRI exams.

The study was performed in three different hospitals, the University Hospitals of Lausanne (CHUV) and Genève (HUG) in Switzerland and the Necker Children’s Hospital in Paris (France), and was approved by the three institutional review boards and ethics committees. The clinical data of the patients included in the study were obtained from the Soarian program (Cerner, London, United Kingdom) at the Swiss hospitals/Mediweb program (Mediweb Solutions, Strasbourg, France) in France and the prenatal imaging exams (US and MRI) from the Archimède program (Archimède Solutions, Geneva, Switzerland) in Switzerland/Astraia program (Astraia Software gmbh, Ismaning, Germany) in France.

### Classification of congenital vascular anomalies

Vascular anomalies were classified according to Mullicken and Glowacki’s classification accepted by the ISSVA [1] (Table 1).

**Table 1:** International Society for the Study of Vascular Anomalies classification of congenital vascular pathologies (adapted from [10])

Tumors	Malformations	
	Simple	Combined malformations
RICH	Capillary malformations	Capillary-venous
		Capillary-lymphatic
NICH	Lymphatic malformations	Lymphatic-venous
		Capillary-lymphatic-venous
PICH	Venous malformations	Capillary-arterial-venous
	Arteriovenous malformations	Capillary-lymphatic-arterial-venous

*NICH* non-involuting congenital hemangioma, *PICH* partially involuting hemangioma, *RICH* rapidly involuting congenital hemangioma

### Prenatal imaging studies

Screening US exams, including color Doppler studies, were usually performed between the 19^th^ and 24^th^ week of pregnancy by obstetricians with a wide experience in prenatal diagnosis with the following US systems: Voluson 730/E8/E10 Expert (GE Healthcare, Strasbourg, France) in Switzerland and France and/or Acuson Sequoia (Siemens Healthineers, Erlangen, Germany) in Switzerland.

Complementary MRI exams were performed using a phased-array multi-channel body coil on a 1.5-tesla (T) MR system with the machines and protocols described in Table 2. MRI exams were evaluated by experienced pediatric radiologists (L.A., P.S., A.-E.M., D.G. and S.H., all with more than 20 years of experience in radiology and more than 15 years of experience in pediatric radiology) who had access to the previous US images and reports.

**Table 2:** Magnetic resonance systems and protocols of fetal magnetic resonance imaging used in the three university hospitals

	Brand	Model	Sequences
Lausanne, Switzerland	Siemens Healthineers (Erlangen, Germany)	Magnetom Symphony	In the 3 fetal planes:
		Aera	T2 half-Fourier acquisition single-shot turbo spin echo (HASTE)
			T2 true fast imaging with steady-state precession (FISP)
			T1-weighted volumetric interpolated breath-hold examination
Geneva, Switzerland	Siemens Healthineers	Avanto	T2 HASTE coronal
			T2 true FISP sagittal
			2-dimensional T1-weighted spoiled incoherent gradient echo sequence axial
Paris, France	GE Healthcare (Waukesha, WI)	Optima MR450w	In the 3 fetal planes:
			Fast imaging employing steady-state acquisition (FIESTA)
			Single-shot fast spin echo
			3-dimensional spoiled gradient echo pulse sequence

Standard prenatal imaging reports included descriptions of the size, volume, anatomical location and organ of origin of the detected pathology. The morphology of the lesion — mostly solid or cystic, micro- or macrocystic, homogeneous or heterogeneous — its characteristics of echogenicity/signal intensity and its effects on the adjacent organs when performed were also included in the reports.

### Review of prenatal studies

The reports of the prenatal imaging studies and their prospectively suggested diagnoses were obtained from the Radoffice (Medspazio, Geneva, Switzerland) and Soarian programs at the CHUV and Geneva and from Carestream (Philips, Horgen, Switzerland) at Necker. They were compared with the final diagnosis resulting from autopsy, anatomopathological exams, surgical reports, and/or postnatal clinical and imaging findings.

The rate of agreement between the prospectively suggested and final diagnoses for both US and MRI were established and the sensitivity for both methods compared. Finally, the clinical relevance of the additional information obtained at MRI and its consequences on management decisions were registered.

### Additional value of MRI studies performed late in pregnancy

In some patients with lymphatic malformations, a second prenatal MRI was performed close to the due date to optimize management upon delivery, with identical protocol to that previously described. The changes in management secondary to the information provided in these late exams were registered and evaluated.

## Results

The retrospective search identified 24 patients who fit the inclusion criteria, with 20 lymphatic malformations and 4 hemangiomas. The mortality rate was 16.7% (4/24 patients). Pregnancy was legally terminated in two fetuses with large lymphatic malformations and two neonates died at birth, one because of an extensive thoracic lymphatic malformation with bilateral pleural effusions and lung infiltration and one because of a rapidly growing hepatic hemangioma causing severe pulmonary hypoplasia.

Data concerning the gender of the fetuses, the anatomical location of the lesions, the main imaging findings, the prenatal suggested diagnosis, the final confirmed diagnosis and the outcome of the patients are described in Table 3 for lymphatic malformations and in Table 4 for congenital hemangiomas. These tables also show the correlation between the prenatal US and MRI and between the prenatal and the final diagnoses.

**Table 3:** Data of the patients with congenital lymphatic malformations included in this series: gender, anatomical location, prenatal suggested diagnosis, final diagnosis and clinical outcome

N	Gender	Anatomical location	US diagnosis	MRI diagnosis	Concordance US/MRI	Imaging findings and complications	Final diagnosis	Concordance pre-/postnatal	Final outcome
1	M	Left face superficial	LM	LM	Yes	- Homogenous fluid content	Mixed LM	Yes	Alive
						- No significant mass effect			
2	F	Left face superficial	LM	LM	Yes	- Homogenous fluid content	Mixed LM	Yes	Alive
						- No significant mass effect			
3	M	Left face + neck	LM	LM	Yes	- Homogenous fluid content	Mixed LM	Yes	Alive
						- Tongue infiltration, tracheal compression, supra-aortic vessels encasement			
4	M	Left face + neck	LM	LM	Yes	- Slightly heterogenous fluid content	Microcystic LM	Yes	Alive
						- No significant mass effect			
5	F	Face + neck	LM	LM	Yes	- Homogenous fluid content	Mixed LM	Yes	Alive
						- No significant mass effect			
6	M	Face + neck	LM	LM	Yes	- Homogenous fluid content	Microcystic LM	Yes	Alive
						- No significant mass effect			
7	M	Neck superficial	LM	LM	Yes	- Homogenous fluid content	Macrocystic LM	Yes	Unknown
						- No significant mass effect			
8	M	Neck	LM	LM	Yes	- Homogenous fluid content	Microcystic LM	Yes	Alive
						- No significant mass effect			
9	M	Supra-hyoid neck	LM	LM	Yes	- Homogenous fluid content	Mixed LM	Yes	Pregnancy termination
						- Tongue infiltration, oropharynx displacement, jugulo-carotid vessels entrapment			
10	M	Right neck superficial	LM	LM	Yes	- Heterogenous fluid content	Macrocystic LM	Yes	Alive
						- No significant mass effect			
11	F	Right neck + thorax	LM	LM	Yes	- Heterogenous fluid content	Macrocystic LM	Yes	Alive
						- Infiltration of the thoracic wall and lung			
12	M	Left neck + thorax	LM	LM	Yes	- Heterogenous fluid content	Macrocystic LM	Yes	Alive
						- Jugulo-carotid vessels encasement			
						- Trachea displacement			
13	M	Right face + neck + thorax	LM	LM	Yes	- Heterogenous fluid content	Mixed LM	Yes	Death at birth
						- Tracheal compression and right lung invasion			
						- Bilateral pleural effusions			
14	M	Right face + neck	LM	LM	Yes	- Homogenous fluid content	Mixed LM	Yes	Pregnancy termination
						- Oropharynx extension			
						- Tongue invasion			
15	M	Right axilla	LM	LM	Yes	- Heterogenous fluid content	Mixed LM	Yes	Alive
						- No significant mass effect			
16	F	Left thorax subcutaneous	LM	LM	Yes	- Homogenous fluid content	Macrocystic LM	Yes	Alive
						- No significant mass effect			
17	F	Right thorax	CPAM	CPAM	Yes	- Homogenous fluid content	Macrocystic LM	No	Alive
						- No significant mass effect			
18	M	Abdomen intraperitoneal	Fetal peritonitis	LM	No	- Homogenous fluid content	Macrocystic LM	Yes	Alive
						- Mass effect without obstruction			
19	M	Abdomen retroperitoneal	Teratoma	Teratoma	Yes	- Heterogenous fluid content, solid aspect	Macrocystic LM	No	Alive
						- No significant mass effect			
20	M	Pelvis presacral	Teratoma	Teratoma	Yes	- Homogenous fluid content	Macrocystic LM	No	Alive
						- No significant mass effect			

*CPAM* congenital pulmonary airway malformation, *LM* lymphatic malformation, *Mixed* macro- and microcystic lesion, *N* patient number

**Table 4:** Data of the patients with congenital hemangioma included in this series: gender, anatomical location, prenatal suggested diagnosis, final diagnosis and clinical outcome

N	Gender	Anatomical location	US diagnosis	MRI diagnosis	Imaging findings and complications	Concordance US/MRI	Final diagnosis	Concordance pre-/postnatal	Final outcome
21	M	Left scalp	Teratoma	Teratoma	- Heterogenous	Yes	Scalp hemangioma	No	Alive
					- No cardiovascular impact				
22	M	Left hepatic lobe	Mesenteric teratoma	Mesenteric teratoma	- Heterogeneous	Yes	Hepatic hemangioma	No	Alive
					- Hypervascular				
					- Enlarged left hepatic vein				
					- Cardiac failure				
23	F	Left hepatic lobe	Hepatic hamartoma	Hepatic hamartoma	- Very heterogenous	Yes	Hepatic hemangioma	No	Death at birth
					- Central cystic areas				
					- Cardiac failure				
					- Hydrops fetalis				
					- Bilateral lung hypoplasia				
24	M	Left hepatic lobe	Hepatic hemangioma	Hepatic hemangioma	- Heterogeneous	Yes	Hepatic hemangioma	Yes	Alive
					- Hypervascular				
					- Enlarged left hepatic vein				
					- Cardiac failure				

*N* patient number

### Congenital lymphatic malformations

**Fig. 1:**
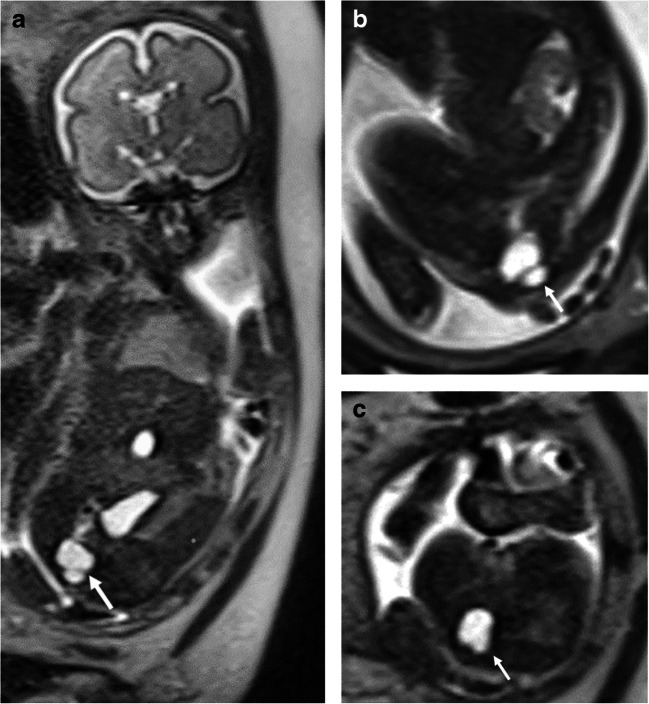
A male fetus at 26 weeks’ gestation with presacral lymphatic malformation (N20, Table 3). **a–c** Coronal oblique (**a**), sagittal paramedian (**b**) and axial (**c**) T2-weighted MR images (repetition time/echo time 1,200/90 ms) show the presacral fluid isointense lesion (*arrows*) with an internal septum. The prenatally suggested diagnosis was a presacral teratoma, based not only on imaging findings but also the typical anatomical location of this common congenital tumor. Pathology after postnatal surgery identified a macrocystic lymphatic malformation

**Fig. 2:**
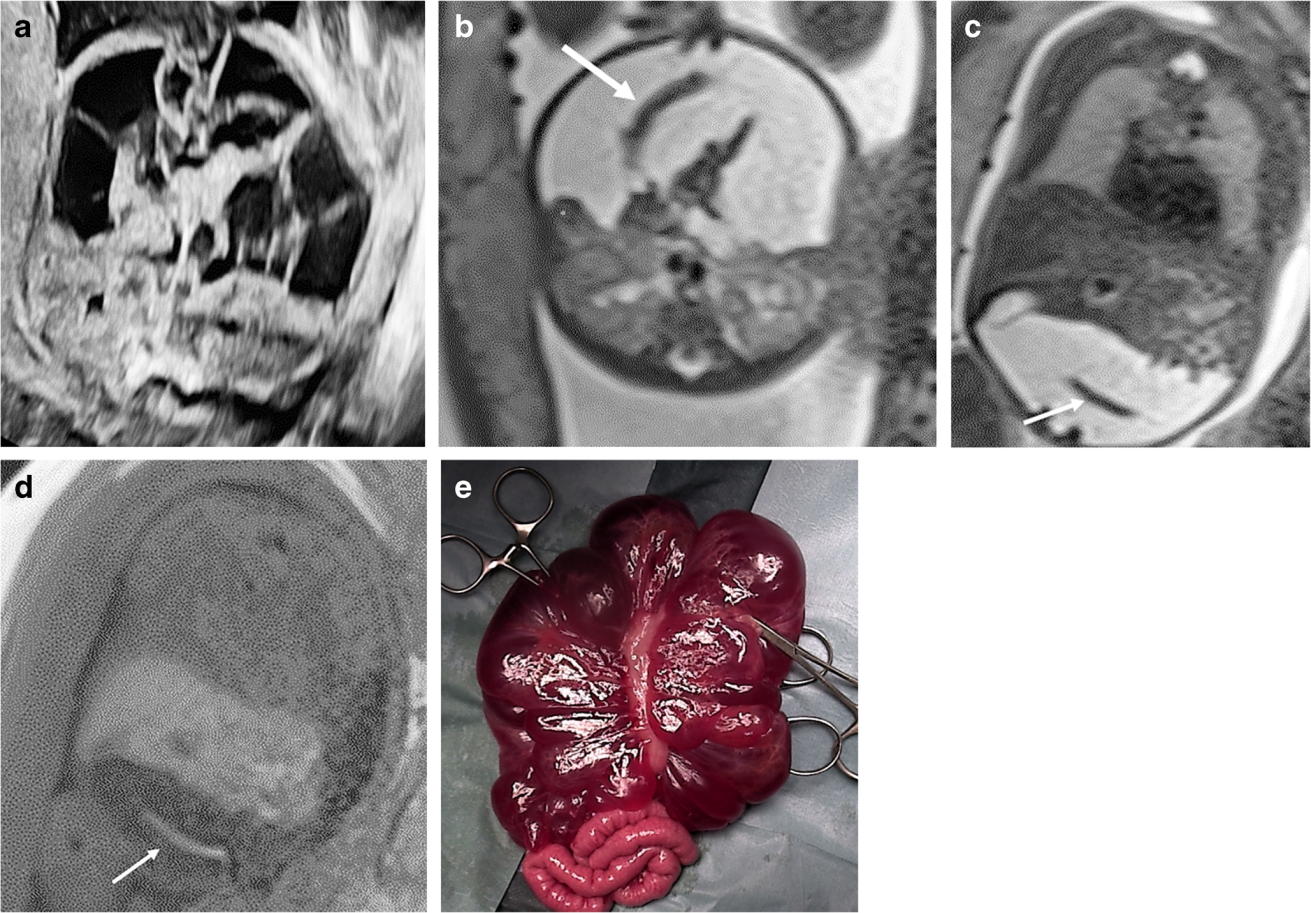
A male fetus at 35 weeks’ gestation with extensive mesenteric malformation and postnatal surgery at 4 months old (N18, Table 3). **a–d** Axial US (**a**) and T2-weighted MR (**b**) images (repetition time [TR]/echo time [TE] 1,200/90 ms) at the same level and coronal T2- (**c**) (TR/TE 1,200/90 ms) and T1-weighted (**d**) (TR/TE 3.29/1.29 ms) MR images show the extensive mesenteric macrocystic lymphatic malformation with homogenous liquid echogenicity and signal intensity and multiple internal septations. The lesion displaces the fetal intraperitoneal organs but shows no significant complication. The fetal colon, surrounded by the lymphatic malformation, is easily identifiable by its meconium filling, hypointense on T2- and hyperintense on T1-weighted images (*arrows*). The lesion was correctly identified only at MRI. **e** Postnatal image during the surgical procedure shows the voluminous lesion surrounding the colon

Lymphatic malformation was suggested in the prenatal imaging of 17 patients and confirmed after birth for the last 3 cases (Table 3). Most of the lesions (14 cases; 70%) were located in the face and neck with occasional extension into the axilla and/or the thorax. The remaining six lesions were located at the axilla (one case), the thorax (two cases), or the abdomen and pelvis (three cases). Prenatal US achieved a correct diagnosis in 16 cases (80%) with the following anatomical locations: cervical and/or facial (11 cases), axillary (1 case), cervicothoracic (3 cases) or thoracic (1 case). Prenatal MRI identified 17 cases (85%), the 16 first previously described at US and 1 additional abdominal lesion. In three fetuses with a postnatally confirmed lymphatic malformation, prenatal US and MRI suggested a teratoma for two abdominal lesions (Fig. 1) and a congenital pulmonary airway malformation (CPAM) for a thoracic lesion (Table 3). The only discrepancy between the suggested diagnosis on US and MRI was an extensive mesenteric lymphatic malformation, which was correctly diagnosed on MRI whereas US suggested a peritonitis after intestinal perforation (Fig. 2).

### Additional value of MRI studies performed late in pregnancy

**Fig. 3:**
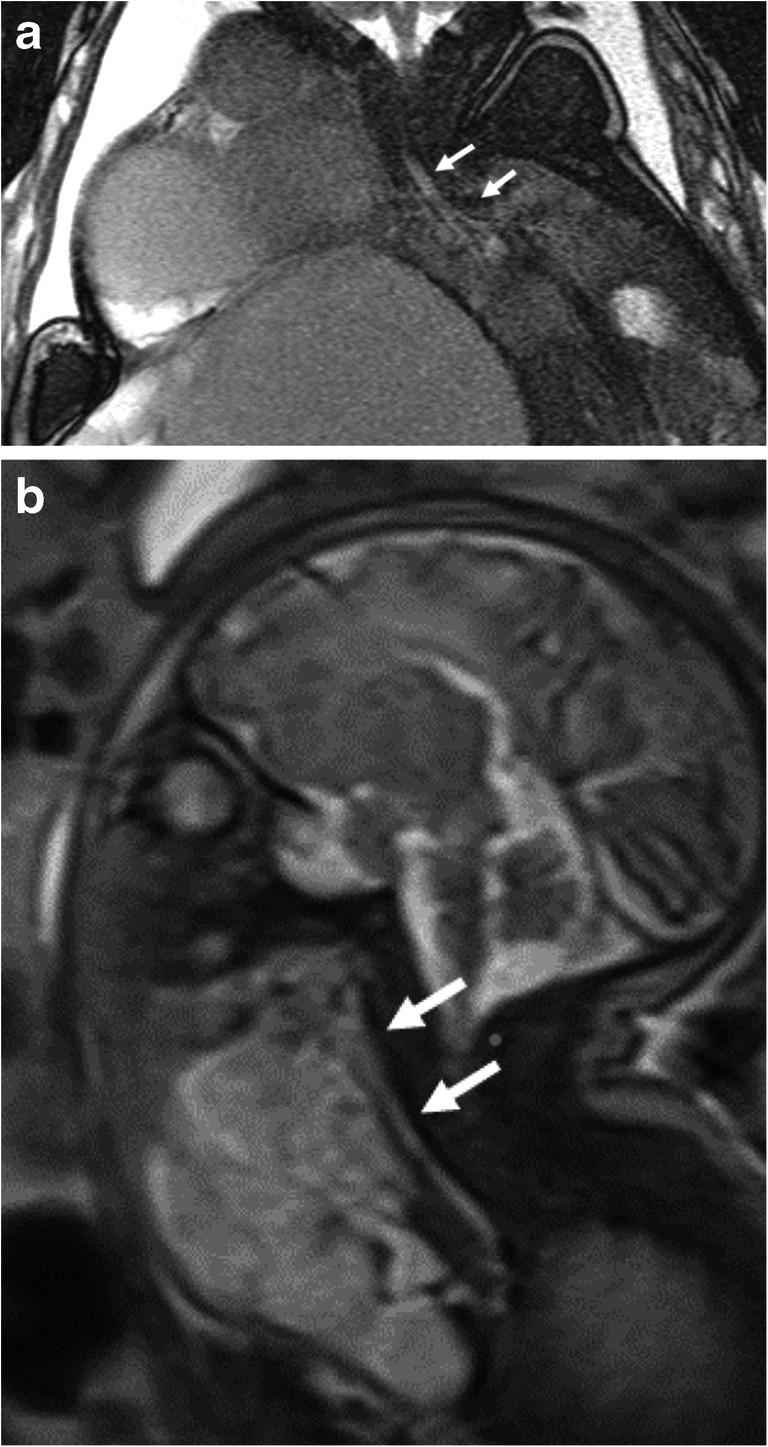
Cervical lymphatic malformations in a female fetus. **a** A coronal T2-weighted MR image (repetition time [TR]/echo time [TE] 6.38/3.19 ms) at 36 weeks’ gestation (N11, Tables 3 and 5) shows the voluminous macrocystic lymphatic malformation with heterogeneous signal intensity after intralesional hemorrhage. The cervical mass extends into the axilla and the thoracic and abdominal wall without invading the fetal organs. Note the normal diameter of the trachea and the main bronchi (*arrows*) and the huge abduction of the fetal arm. **b** In opposite, the midline sagittal T2-weighted image (TR/TE 1,200/89 ms) at 35 weeks’ gestation (N3, Tables 3 and 5) shows the anterior cervical lymphatic micro- and macrocystic malformation, extending into the submental space without invading the tongue. Note the resulting compression of the larynx and the trachea (*arrows*). An ex utero intrapartum treatment was performed at birth

In five fetuses with prenatally diagnosed lymphatic malformations, a second MRI exam was performed during advanced pregnancy (33–36 weeks). Indication for these late studies were an anatomical location close to the upper respiratory airways (five cases) and/or a voluminous lesion (two cases) (Fig. 3). These exams were performed for management at birth, including decisions about the type of delivery and the need for extracorporeal intrapartum treatment (EXIT procedure) [11]. Table 5 includes the additional information provided by these late performed MRIs and its influence on patient management.

**Table 5:** Additional information provided by late pregnancy MRI exams in patients with lymphatic malformations

N	Location	Time of MRI (weeks of pregnancy)	Additional information at 2nd MRI	Management
3	Left face + neck	32/35	- Major volume augmentation without hemorrhage	- EXIT procedure
				- Emergency cesarean at 36 weeks of pregnancy
			- Oropharynx distortion	
			- Increasing tracheal compression	
5	Face + neck	26/34	- No significant tracheal displacement	- Normal birth
			- No hemorrhage	
11	Right neck + thorax	25/36	- Major volume augmentation due to hemorrhage	- No need of EXIT
				- Elective cesarean at 37 weeks of pregnancy
			- Slight lateral tracheal displacement	- Immediate intubation
			- Extreme arm abduction	
12	Left neck + thorax	22/34	- Mediastinal extension	- No need of EXIT
			- Slight tracheal displacement	- Elective cesarean at 39 weeks of pregnancy
			- No hemorrhage	- Immediate intubation
18	Abdomen intraperitoneal	26/35	- Major volume augmentation without hemorrhage	- Elective cesarean at 38 weeks of pregnancy
			- Increasing abdominal distention	- Surgical excision at 2 months of age
			- No intestinal obstruction	

*EXIT* ex utero intrapartum treatment, *N* patient number

### Congenital hemangiomas

**Fig. 4:**
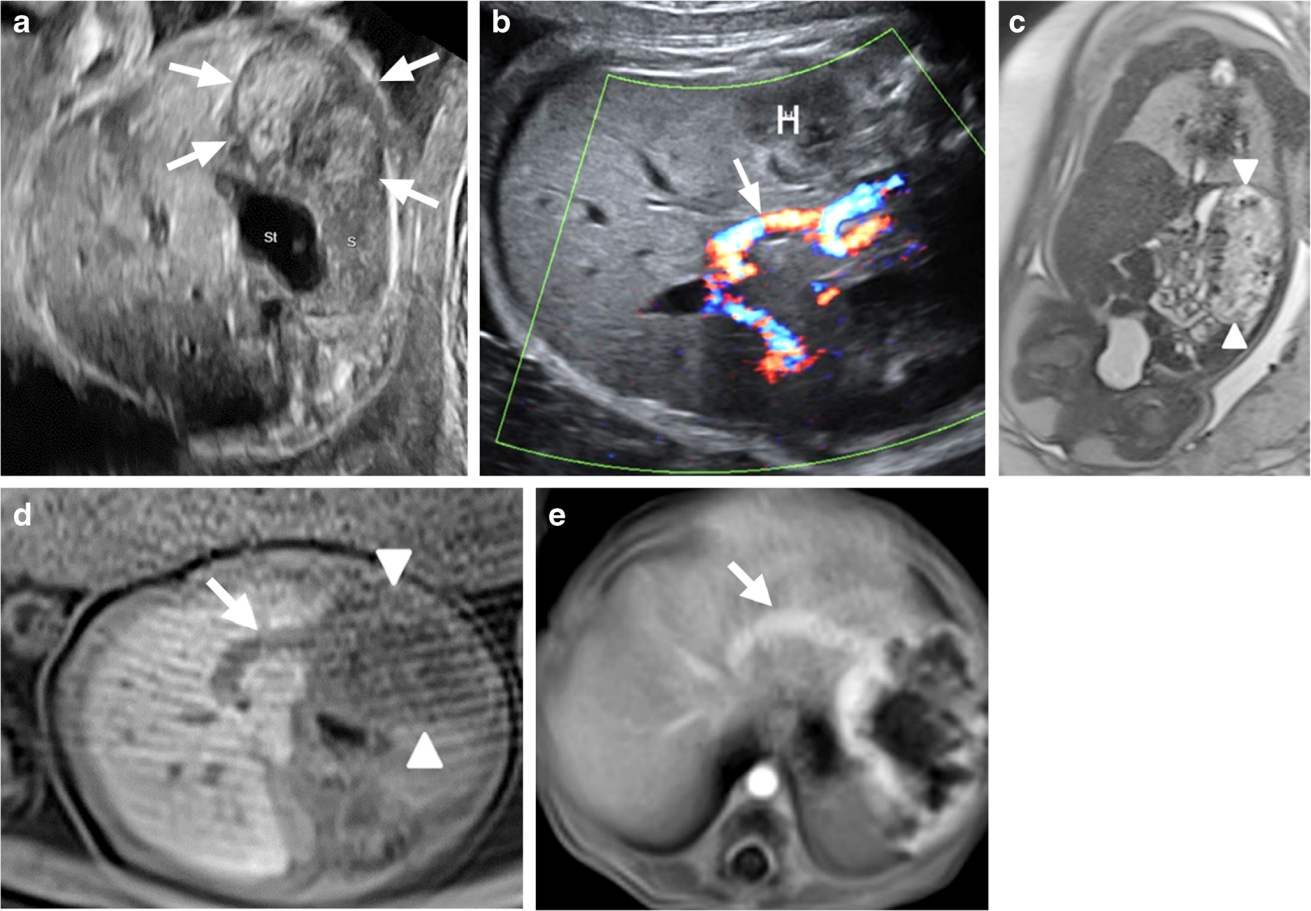
A male fetus at 33 weeks’ gestation (N22) with hepatic hemangioma and postnatal images at 4 days old. **a** Transverse US image shows a voluminous, solid appearing, heterogeneous left hepatic mass. *Arrows* indicate the borders of the lesion. *S* spleen, *St* stomach. **b–d** Axial Doppler US image (**b**), coronal T2-weighted (**c**) (repetition time [TR]/echo time [TE] 1,000/92 ms) image at the same level as well as axial T2-weighted (**d**) MR image (TR/TE 6.60/3.30 ms) show the inhomogeneous, exophytic growing lesion arising from the left hepatic lobe (*H* in **b**, *arrowheads* in **c**, **d**). Note the numerous tubular forming flow voids indicating vascular structures (*arrow* in **d**) and the extreme enlarged left hepatic vein (*arrow* in **b**). **e** Postnatal T1-weighted MR image (TR/TE 3.61/1.61 ms) after contrast shows the marked early peripheral enhancement of the lesion at the arterial phase and confirms the enlarged left hepatic vein (*arrow*). After significant regression, the tumor was resected when the boy was 18 months old

**Fig. 5:**
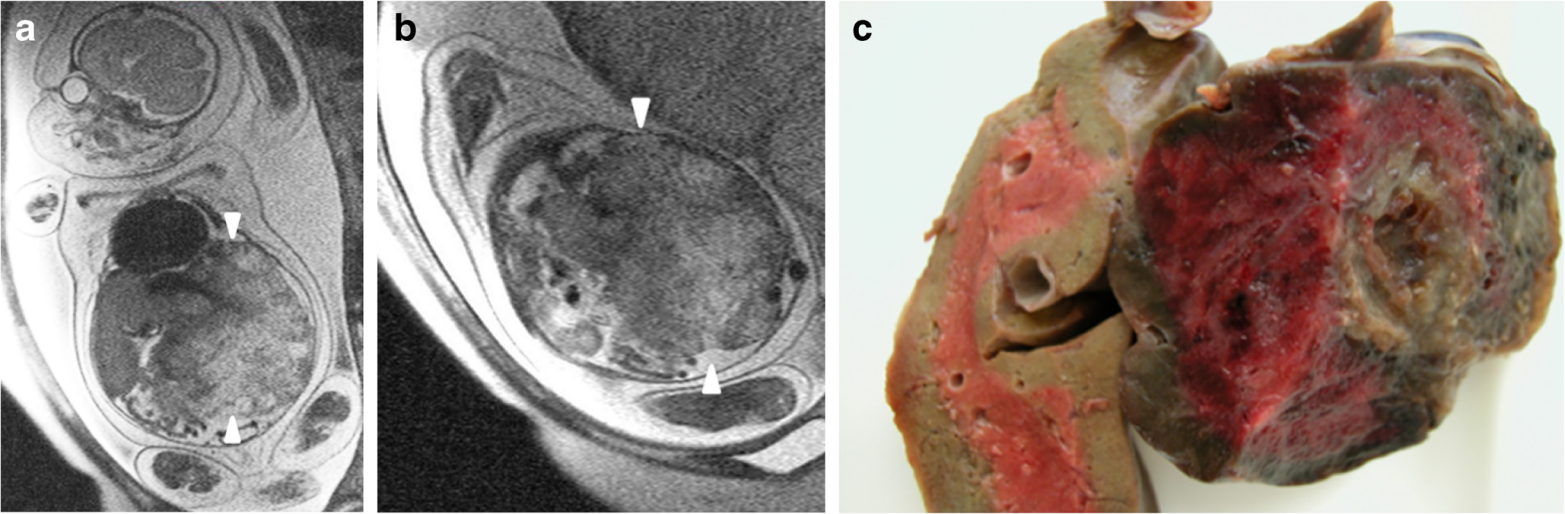
A male fetus at 27 weeks’ gestation with hepatic hemangioma (N23). **a–b** Coronal (**a**) and left sagittal (**b**) T2-weighted MR images (repetition time/echo time 1,530/86 ms) show a huge, heterogeneous left abdominal mass arising from the left hepatic lobe. Note the absence of tubular intralesional structures. *Arrowheads* show the borders of the lesion. The tumor causes bilateral elevation of the hemidiaphragms and severe secondary lung hypoplasia, fetal cardiomegaly and marked hydrops fetalis. The patient died at birth after an emergency cesarean at the 27th week of pregnancy. **c** Macroscopic view of the fetal liver confirms the hepatic origin of this tumor and reveals central cavities and extensive necrosis

The anatomical location of the four confirmed congenital hemangiomas included in this series were the scalp (one case) and the liver (three cases) (Figs. 4 and 5). The prenatal accuracy of diagnosis was very low and showed no difference between the two imaging methods, with only 1 of 4 (25%) confirmed hemangiomas correctly identified in utero (Table 4).

## Discussion

The ISSVA acceptance of Mullicken and Glowacki’s classification for congenital vascular anomalies [1] has contributed considerably to a better understanding of these pathologies while the generalization of screening US has increased their in utero detection rate. Impressive technical advances and an increased availability of MRI have led to an extension of this method for diagnosing prenatal pathology. However, data concerning its real contribution for congenital vascular anomalies remain limited [12–15].

### Congenital lymphatic malformations

Lymphatic malformation is the most often detected vascular malformation in the fetus and the most widely described in the literature [4, 15–18]. The reported incidence is 1:2,000–6,000 cases in live births, with a slight male predominance [16]. They can occur in any location, but are much more often located in the neck (75%) and the axilla (20%) than in the abdomen (2%), limbs (2%) and mediastinum (1%) [4, 15, 17]. They are usually classified as microcystic (cysts <2 cm in size), macrocystic (>2 cm) and mixed lesions. Macrocystic lesions are usually more voluminous and therefore easier to detect in utero than microcystic ones [17]. At US, these malformations appear as multiseptated cystic lesions, lacking solid components, vascularity and calcifications. However, a recent review has described a greater imaging variability than previously reported, including mixed cystic/solid lesions and occasional calcifications [17].

Our series evaluated 20 confirmed lymphatic malformations and obtained a high accuracy in the detection and correct identification of these lesions on both prenatal US and MRI, 80% and 85%, respectively. All our cervicofacial and axillary located malformations were correctly identified on both methods and compared to previously published series [4, 15, 17] we had a higher percentage of rare anatomical locations, including the thorax (2 cases; 10%) and the abdomen and/or pelvis (3 cases; 15%). We observed a significant discordance between the prenatal suggested diagnosis and the final diagnosis in these uncommon locations. US identified only one of two thoracic and none of three abdominal malformations, whereas MRI identified one thoracic and one abdominal lesion. For the two remaining unidentified cases of abdominal lesions, both methods suggested the teratoma, probably influenced by the presacral location of one of these lesions, typical for this common fetal tumor (Fig. 1).

Atypical imaging findings were the second explanation for the discordance between the prenatal and the final diagnosis. As previously reported, the differentiation between a lymphatic malformation and a fetal teratoma can be extremely difficult in the presence of calcifications, hemorrhage or of mixed cystic and solid lesions [17] Indeed, one of the misinterpreted mesenteric lymphatic malformations showed an extensive hemorrhage with marked heterogenicity in utero.

### Additional information provided by MRI

MRI is less affected than US by fetal position, oligohydramnios, maternal obesity and fetal bone superposition and can more precisely determine the extent of a lesion and its relationship to the adjacent structures [16, 18, 19]. In our series, the information provided by complementary MRI improved the echographic suggested diagnosis and changed the management of 2 (10%) patients. In a thoracic lymphatic malformation, MRI showed an extension toward the mediastinum and into the lungs in addition to voluminous pleural effusions already detected on US, which led to a pregnancy continuation with only comfort care at birth. In a second case, MRI correctly identified a voluminous mesenteric lymphatic malformation and excluded relevant complications, which led to the decision to continue the pregnancy (Fig. 2).

### MRI studies performed late in pregnancy

In cervically located lymphatic malformations, MRI can document the extension toward the mediastinum and evaluate the proximity to the brachial plexus [16, 18] or the degree of compression of the airways [11, 18, 19], information difficult to obtain by US alone [13]. A second MRI exam at advanced pregnancy performed in five lymphatic malformations located in a sensitive anatomical location provided accurate anatomical information and enabled decisions about the type of delivery (Table 5), including the successful performance of an EXIT procedure in a fetus showing increasing tracheal compression (Fig. 3).

### Congenital hemangiomas

Congenital hemangiomas are already fully developed at birth [20]. RICH and NICH types have an almost equal gender distribution, are usually solitary lesions and have a predilection for the skin, mostly in the head or limbs. However, the reported imaging findings of these lesions are still limited, with descriptions of isolated cases, concerning mainly RICH-type lesions in typical locations [5, 12, 21].

Hemangiomas are the most frequently detected liver tumors in fetuses and neonates, concerning more than 60% of all congenital hepatic lesions [22]. They are often heterogeneous on US and may contain identifiable calcifications. On MRI, they also are frequently heterogeneous, with foci of hyperintensity on T1-weighted images and hypointensity on T2-weighted images and intratumoral flow void that represents tubular vascular structures. In a series of 16 children, Franchi-Abella et al. [23] reported well-defined, heterogeneous, hypoechoic lesions compared to the normal liver on US with intratumoral abnormal vessels and enlarged hepatic arteries and/or veins. On MRI, they showed a high signal intensity on T2-weighted images and a low signal intensity on T1-weighted images when compared to the normal liver, with intralesional signal flow voids. Jiao-Ling et al. [24] described well-defined masses on US exams in a series of six congenital hepatic hemangiomas that were mostly hypoechogenic compared to the normal liver with significant rates of heterogeneity, necrosis, cystic cavities and calcifications. Color Doppler showed enlarged hepatic arteries and tortuous, dilated veins. The lesions were hypointense on T1-weighted MR images and hyperintense on T2-weighted images compared to the normal liver.

In our series, the sensitivity of prenatal US and MRI for congenital hemangiomas was only 25%, and showed no difference between both prenatal methods. Only one of three hepatic hemangiomas was identified in utero*.* (Table 4). Strangely, the three hepatic tumors were exophytic, growing from the edge of the left lobe. Two of them had almost identical imaging findings and were hypoechogenic, heterogeneous on US and mostly hyperintense on T2-weighted MR images compared to the normal liver with clearly visible flow voids and an extremely enlarged left hepatic vein (Fig. 4). These two lesions were classified as RICH tumors according to the postnatal evolution. In contrast, the third hemangioma was a rapidly growing, heterogeneous lesion with extensive avascular areas on US Doppler and no intratumoral flow voids identifiable on MRI. Autopsy after emergency cesarean and death at birth revealed a huge hemangioma with extensive areas of hemorrhage, thrombosis and necrosis (Fig. 5).

### Additional information provided by MRI

Complementary MRI influenced the management in a rapidly growing hepatic congenital hemangioma while revealing significant bilateral pulmonary hypoplasia. Emergency cesarean was performed, but the patient died at birth from combined cardiorespiratory insufficiency.

Franchi-Abella et al. [23] suggested that fetal US should remain the standard diagnostic method for hepatic hemangiomas and proposed that MRI should only be performed when the diagnosis is unclear or the lesion is not well-delimited. In opposite, Jiao-Ling et al. [24] and our own results show that voluminous hepatic hemangiomas often have a great imaging variability that could explain the low rates of echographic diagnosis in utero. Although our prenatal rate remained low after MRI, we still believe these exams helped evaluate the effects of the lesions on the fetus.

This article increases the limited available information regarding the prenatal imaging findings of the most frequently detected congenital vascular anomalies and reveals the main difficulties in their prenatal diagnosis. However, it has some limitations, including its retrospective character and a reduced number of patients despite the participation of three university hospitals. Therefore, these results should be confirmed by more extensive and prospective studies.

## Conclusion

Macrocystic lymphatic malformations and rapidly involuting congenital hemangioma are the most commonly detected congenital vascular anomalies in utero. A rare anatomical location and atypical imaging findings, such as hemorrhage or necrosis, complicate their prenatal diagnosis. The main role of complementary MRI is probably not to improve the diagnosis but to provide high-defined anatomical data to guide the management and anticipate complications at birth.
